# A Simple and Efficient Method for Assembling TALE Protein Based on Plasmid Library

**DOI:** 10.1371/journal.pone.0066459

**Published:** 2013-06-20

**Authors:** Zhiqiang Zhang, Duo Li, Huarong Xu, Ying Xin, Tingting Zhang, Lixia Ma, Xin Wang, Zhilong Chen, Zhiying Zhang

**Affiliations:** College of Animal Science and Technology, Shaanxi Key Laboratory of Molecular Biology for Agriculture, Northwest A&F University, Yangling, Shaan'xi, P. R. China; Florida State University, United States of America

## Abstract

DNA binding domain of the transcription activator-like effectors (TALEs) from *Xanthomonas sp.* consists of tandem repeats that can be rearranged according to a simple cipher to target new DNA sequences with high DNA-binding specificity. This technology has been successfully applied in varieties of species for genome engineering. However, assembling long TALE tandem repeats remains a big challenge precluding wide use of this technology. Although several new methodologies for efficiently assembling TALE repeats have been recently reported, all of them require either sophisticated facilities or skilled technicians to carry them out. Here, we described a simple and efficient method for generating customized TALE nucleases (TALENs) and TALE transcription factors (TALE-TFs) based on TALE repeat tetramer library. A tetramer library consisting of 256 tetramers covers all possible combinations of 4 base pairs. A set of unique primers was designed for amplification of these tetramers. PCR products were assembled by one step of digestion/ligation reaction. 12 TALE constructs including 4 TALEN pairs targeted to mouse Gt(ROSA)26Sor gene and mouse Mstn gene sequences as well as 4 TALE-TF constructs targeted to mouse Oct4, c-Myc, Klf4 and Sox2 gene promoter sequences were generated by using our method. The construction routines took 3 days and parallel constructions were available. The rate of positive clones during colony PCR verification was 64% on average. Sequencing results suggested that all TALE constructs were performed with high successful rate. This is a rapid and cost-efficient method using the most common enzymes and facilities with a high success rate.

## Introduction

Efficient targeted genome editing relies on the use of engineered nucleases, artificial proteins composed of a customizable sequence-specific DNA-binding domain fused to a nuclease that cleaves DNA in a non-sequence-specific manner. The technology platform heavily relies on engineering sequence-specific DNA-binding domain. TALEN is a newly developed technology in targeted genome editing following the zinc finger nuclease (ZFN) technology [1]. Transcription activator-like effectors (TALEs) from the genus *Xanthomonas* consist of a series of novel DNA-binding proteins [2], [3]. Deciphering the DNA binding mechanism of TALE repeats opens a new avenue to develop TALE-based technology for genome editing [4], [5]. The sequence binding specificity of TALE is determined by highly conserved tandem repeats in the central DNA-binding domain. Naturally occurring repeats in TALE consist of 33–35 amino acids with two variable di-residues (RVDs) at positions 12 and 13 specifying one of the four bases together [4], [5]. TALE repeats can be adjacent in arrays of custom length with capability to target specific DNA sequences. Artificial TALE transcription factors (TALE-TFs) or TALE nucleases (TALENs) have been constructed by fusing customized repeat arrays to transcriptional activation domain or FokI cleavage domain [6], [7]. Customized TALE-TFs have efficiently up-regulated the expression of endogenous human Sox2, Klf4 genes [8] and mouse Oct4 gene [9], which might provide a way for pluripotent stem cells induction. Genome alterations have been generated by repair of DNA double-strand breaks (DSBs) through non-homologous end-joining (NHEJ) or homologous recombination (HR) induced by customized TALENs in plants [6], yeasts [10], zebrafish [11], [12], [13], *Xenopus* embryos [14], rat embryos [15] and human somatic [16], [17] and pluripotent stem cells [18].

Few researchers chose to use commercial synthesized TALENs for their studies [16]. Although many assembly methods have been recently reported, including a series of methods derived from Golden Gate cloning [6], [8], [10], [19], [20], [21], [22], regular cloning methods [12], [23] and high-throughput methods [24], [25], a rapid, convenient, and more cost-efficient method with high success rate is desired by researchers who are interested in TALE application.

Here we describe a rapid, convenient and cost-efficient method with high success rate based on Golden Gate cloning strategy. Our results indicate that this is a feasible method which may make TALEN and TALE-TF construction more universal.

## Materials and Methods

### Molecular Biology Reagents

Restriction enzymes and T4 DNA ligase used in this study were purchased from New England Biolabs (NEB, USA). Taq and Pfu DNA polymerase were bought from Transgen Biotech (Beijing, China). pGEM-T easy vector was bought from Tiangen Biotech (Beijing, China). 4 monomer fragments (NI, HD, NG and NN), 4 pLenti-EF1a-Backbone plasmids (backbone plasmids for TALE-TF construction and expression) and pST1374 vector (backbone plasmids for TALEN construction and expression) were bought from Addgene (USA).

Plasmid preparations were performed by using Plasmid Mini Kit (Omega, USA) and DNA gel extractions by using AxyPrepTM DNA Gel Extraction Kit (Axygen, USA) following the manufacturer’s protocol. Plasmid DNA concentration was measured using a NanoDrop 2000 Spectrophotometer (Thermo, USA).

### Construction of Monomer Plasmids

Optimized four monomers (NI, HD, NN and NG) with minimized repetitiveness were used as template for monomer plasmid construction as previously described [8]. Each monomer was amplified with four pairs of primers TALE-F1/TALE-R1, TALE-F2/TALE-R2, TALE-F3/TALE-R3 or TALE-F4/TALE-R4. Each pair of primers (Table 1) was designed such that each monomer had four possible cohensive ends as TGAC/ACTC, ACTC/CCTC, CCTC/ATTA, and ATTA/CTTA at 5′/3′ ends after *Bsa*I digestion [8]. The amplified monomers were ligated into pGEM-T easy vector after purification. Transformed cells were plated on LB solid medium containing 100 ug/ml ampicillin, with X-gal and IPTG for blue/white screening of recombinants. White colonies were picked and 37°C overnight cultured in LB medium with 100 ug/ml ampicillin. Monomer plasmids were sequenced with T7 forward primer (Table 2) on pGEM-T easy vector. Therefore, the monomer library of 16 monomers was constructed.

**Table 1:** Primer sequences for monomer amplification.

Primer name	Sequence
TALE-F1	5′-ATATAGATGCCGTCCTAGCGcgtctcCTGACCCCAGAGCAGGTCGTGG-3′
TALE-F2	5′-TGCTCTTTATTCGTTGCGTCggtctcGACTCACCCCAGAGCAGGTCGTG-3′
TALE-F3	5′-TGCTCTTTATTCGTTGCGTCggtctcGCCTCACCCCAGAGCAGGTCGTG-3′
TALE-F4	5′-TGCTCTTTATTCGTTGCGTCggtctcGATTAACCCCAGAGCAGGTCGTG-3′
TALE-R1	5′-TCTTATCGGTGCTTCGTTCTggtctcTGAGTCCGTGCGCTTGGCAC-3′
TALE-R2	5′-TCTTATCGGTGCTTCGTTCTggtctcTGAGGCCGTGCGCTTGGCAC-3′
TALE-R3	5′-TCTTATCGGTGCTTCGTTCTggtctcTTAATCCGTGCGCTTGGCAC-3′
TALE-R4	5′-AAGTATCTTTCCTGTGCCCAcgtctcTTAAGCCGTGCGCTTGGCAC-3′

**Table 2:** Primer sequences for sequencing, subcloning and PCR verification.

Primer name	Sequence
T7	5′-TAATACGACTCACTATAGG-3′
SP6	5′-ATTTAGGTGACACTATAG-3′
N-F	5′-CCTCTAGAATGTCGCGGACCCGGCTCCC-3′
C-R	5′-CGCGGATCCGCCGAGGCAGGCCAGCGCTAC-3′
SeqF	5′-CATGAGGCGATCGTCGGTGT-3′
SeqR	5′-AGTGAGTGCGGCCAGCGC-3′

### Construction of Tetramer Plasmids

Tetramer plasmids were constructed using cut/ligation reaction based on Golden Gate cloning strategy, taking 16 monomer plasmids as building blocks. A cut/ligation reaction was set up in a 20 ul reaction containing 100 ng of each 4 monomer plasmids, 1 ul *Bsa*I (10 U/ul), 1 ul T4 DNA ligase (400 U/ul), 2 ul 10 × T4 DNA ligase buffer and distilled water. The reaction was performed in a thermocycler with the following program: 5 min at 37°C, 5 min at 16°C for 35 cycles followed by 2 h at 16°C and 20 min at 80°C.

3 ul reaction mixture was transformed into *E. coli* DH5αand the cells were plated on LB solid medium containing 100 ug/ml ampicillin. Plates were incubated overnight at 37°C. Colony PCR were used for verification with primer T7 (forward primer annealing on pGEM-T vector ∼60 bp before tetramer) and SP6 (reverse primer annealing on vector ∼80 bp after tetramer). The positive clone should have a single band of size 641 bp. Positive clones were grown in LB liquid medium with 100 ug/ml ampicillin overnight. Plasmids were extracted the next day. Tetramer plasmids were identified by sequencing with T7 forward primer. By repeating the steps above, we constructed a library of 256 tetramer plasmids.

### Construction of TALEN Expression Backbones

The regions including N-terminal, two *Bsm*BI sites, a 0.5 repeat (coding half of repeat NI, HD, NG or NN) and truncated C-terminus of TALE gene from each of the four pLenti-EF1a-Backbones were amplified by PCR with forward primer N-F and reverse primer C-R (Table 2). The *Xba*I and *Bam*HI restriction sites were added at 5′ end of the primers respectively. The PCR products were cloned into pST1374 vector, designated as pST-TALEN-Backbones. The sequence fidelity was verified by sequencing with T7 forward primer.

### Construction Protocol for TALEN or TALE-TF Constructs

**Figure 1:**
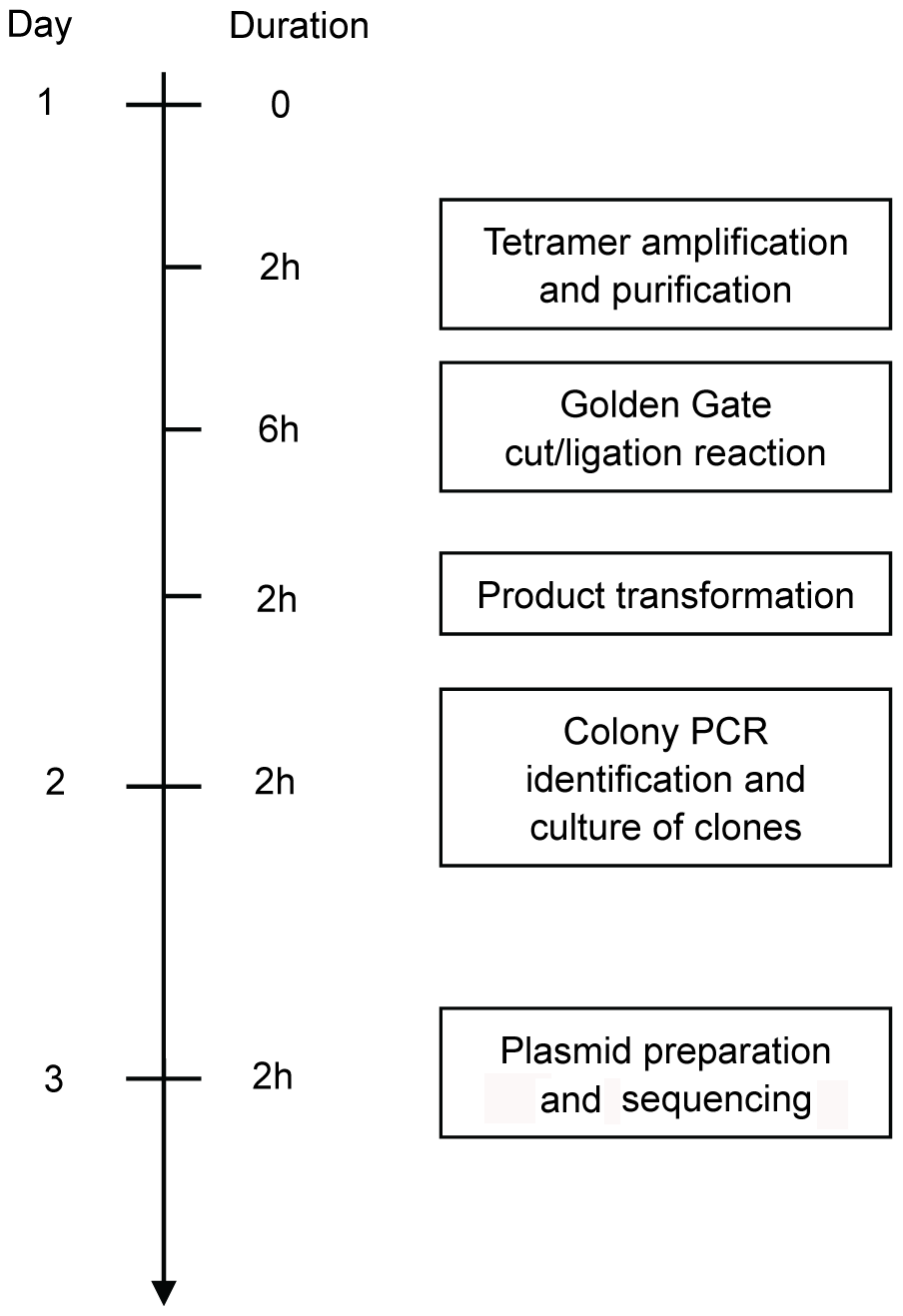
Timeline for the construction of TALE constructs. Steps of the construction of TALENs and TALE-TFs are presented. TALE constructs can be generated in 3 days following the described steps. Samples of each step can be stored at −20°C for further use.

Assembly of a customer TALE-TF or TALEN construct took about 3 days based on our tetramer library. A schematic representation is shown in Figure 1. The protocol for construction of a customized TALEN with 12.5 tandem repeats is summarized as follows:

#### Day 1 AM

Tetramer plasmids containing repeats 1–4, 5–8 and 9–12 were amplified with primer pairs Tetramer-Fv\Tetramer-R1, Tetramer-F2\Tetramer-R2 and Tetramer-F3\Tetramer-Rv, respectively. Sequences of primers are given in Table 3. Typically, the PCR reaction was prepared to a total volume of 100 ul consisting of 40 ng tetramer plasmids, 10 ul of 10 mM dNTP (2.5 mM each), 2 ul of forward primer (5 uM) and 2 ul reverse primer (5 uM), 1 ul Taq DNA polymerase (5 U/ul), 10 ul of 10 × Taq polymerase buffer. PCR reactions were performed using the following program: 95°C for 4 min; 94°C for 30 sec, 57°C for 30 sec, 72°C for 35 sec, cycle for 30 times; 72°C for 10 min.

**Table 3:** Primer sequences for TALE repeats amplification.

Primer name	Sequence
Tetramer-Fv	5′-ATATAGATGCCGTCCTAGCGcgtctcCTGACCCCAG-3′
Tetramer-R1	5′-AAGTATCTTTCCTGTGCCCAcgtctcTTAAGCCGTGC-3′
Tetramer-F2	5′-ATATAGATGCCGTCCTAGCGcgtctcGCTTAACCCCAG-3′
Tetramer-R2	5′-AAGTATCTTTCCTGTGCCCAcgtctcTGAGCCCGTGC-3′
Tetramer-F3	5′-ATATAGATGCCGTCCTAGCGcgtctcGGCTCACCC-3′
Tetramer-Rv	5′-AAGTATCTTTCCTGTGCCCAcgtctcTGAGTCCG-3′
Tetramer-R3	5′-AAGTATCTTTCCTGTGCCCAcgtctcTAAGACCGTGC-3′
Tetramer-F4	5′-ATATAGATGCCGTCCTAGCGcgtctcGTCTTACCCCAG-3′
Tetramer-R4	5′-AAGTATCTTTCCTGTGCCCAcgtctcTCAATCCGTGC-3′
Tetramer-F5	5′-ATATAGATGCCGTCCTAGCGcgtctcGATTGACCCCAG-3′
Tetramer-R5	5′-AAGTATCTTTCCTGTGCCCAcgtctcTTAGCCCGTGC-3′
Tetramer-F6	5′-ATATAGATGCCGTCCTAGCGcgtctcGGCTAACCCCAG-3′
Tetramer-R6	5′-AAGTATCTTTCCTGTGCCCAcgtctcTCAGCCCGTGC-3′
Tetramer-F7	5′-ATATAGATGCCGTCCTAGCGcgtctcGGCTGACCCCAG-3′
RDT-R2	5′-AAGTATCTTTCCTGTGCCCAcgtctcTAAGCCCGTGC-3′
RDT-F3	5′-ATATAGATGCCGTCCTAGCGcgtctcGGCTTACCC-3′

The PCR product was run on 2% (wt/vol) agarose gel at 20 V cm^−1^ for 30 min or longer until all bands were completely separated. The correct amplified tetramer was about 465 bp in size. The tetramer amplicon was purified with PCR purification kit and finally eluted in 30 ul of distilled water. The purified tetramers (100 ng each) with the expression vector pST-TALEN-Backbone (200 ng) were subjected to a cut/ligation reaction in a 10 ul volume containing 1 ul of *Bsm*BI (10 U/ul), 1 ul of T4 DNA ligase (400 U/ul), 1 ul of 10×T4 DNA ligase buffer and distilled water. The cut/ligation reaction was performed with cycles in thermocycler. The digestion with *Bsm*BI was carried out at 42°C for 5 min and ligation at 16°C. The reaction was repeated for 30 cycles. Although the optimal temperature of *Bsm*BI is 55°C, its activity at 42°C is adequate for the cut/ligation reactions (*Bsm*BI still maintains 20% cleavage activity at 37°C), as higher temperature will accelerate inactivity of DNA ligase.

#### Day 1 PM

3 ul of the cut-ligation reaction mixture was transformed into 40 ul competent *E. coli* strain DH5α. Cells were plated on LB solid medium containing 100 ug/ml ampicillin and incubated overnight at 37°C.

#### Day 2

Typically, hundreds to thousands of colonies would grow up on the plates against few on the negative control. 20 colonies were then picked out for colony PCR verification. Primers SeqF (forward primer annealing at the last of the N-terminal on backbone) and SeqR (reverse primer annealing at the beginning of the C-terminal on backbone) were used for amplification. Their sequences are listed in Table 2. PCR reactions were made in 20 ul reaction containing 1 ul colony suspension, 2 ul 10 mM dNTP (2.5 mM each), 0.4 ul forward primer (5 uM) and 0.4 ul reverse primer (5 uM), 0.2 ul Taq DNA polymerase (5 U/ul) and 2 ul 10 × Taq DNA polymerase buffer. The reaction was performed in a thermocycler with the following program: 95°C for 4 min; 94°C for 30 sec, 56°C for 30 sec, 72°C for 1 min 45 sec, cycle for 30 times; 72°C for 10 min.

PCR results were verified on a 1% (wt/vol) agarose gel. For a complete insert of 12 monomers (three tetramers ligated into the TALE backbone vector) together with a half one on the backbone, the product should be a single band of 1535 bp in size. The clones with the correct band were then cultured into 3 ml of LB medium with 100 ug/ml ampicillin and shook at 37°C overnight.

#### Day 3

The plasmids were prepared by using Plasmid Mini Kit following the manufacturer’s protocol and verified by *Xba*I/*Bam*HI restriction digestion before sending for sequencing with primers SeqF and SeqR.

## Results

### Construction of a Tetramer Library

**Figure 2:**
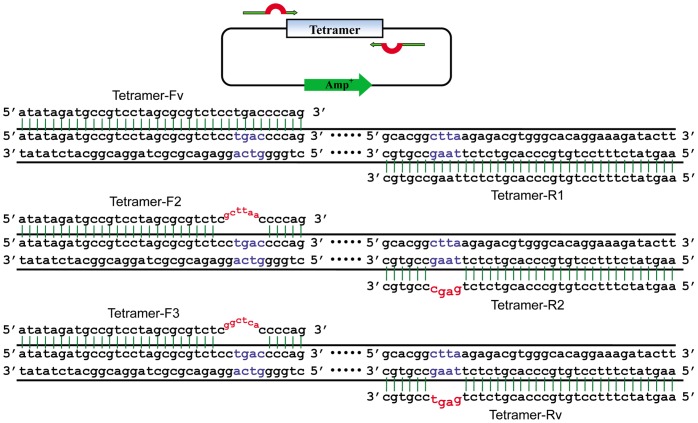
Schematic representation of tetramer PCR mutation. Cohesive ends of tetramers in the library can be changed after amplification with Tetramer-F1/Tetramer-R1, Tetramer-F2/Tetramer-R2 or Tetramer-F3/Tetramer-R3 for position specification. The original bases on tetramer plasmids are in blue and mismatches on primers are in red.

We previously assembled a TALEN pair targeting human CCR5 gene by following the described method [8] into our pST-TALEN-Backbones and found that it is not only time consuming but also has low fidelity. This prompted us to consider building tetramer library which covers all 256 possible combinations binding 4 nucleotides. This library would be used as building blocks for assembling TALE proteins. The repeat tetramers in the library are all flanked by *Bsm*BI sites, which will generate TGAC at 5′ end and CTTA at 3′ end after digestion. To generate different cohesive ends for assembling tetramers, we designed the primers tagged with *Bsm*BI site, which yielded flexible cohesive ends as needed and were used to amplify the tetramers (Figure 2). The first pair of primers used to amplify the first tetramer generated a cohesive end at 5′ end compatible with the 5′ end from vector digestion and at 3′ end compatible with the 5′ end of the second tetramers amplified with the second pair of primers. The second pair of primers was designed to generate the tetramer at 5′ end compatible with the 3′ end of the first tetramer and at 3′ end compatible with the 5′ end of the third tetramer, and so on. Theoretically, we can assemble a TALE protein containing up to 30 monomers at once.

To efficiently construct tetramer library, we first generated a monomer library containing 16 monomer plasmids. The 4 pairs of unique primers were designed such that the amplified monomers can be ligated into any chosen position (such as position 1, or 2, or 3 and 4) in tetramers. Four monomers recognizing 4 DNA bases (NI = A, HD = C, NG = T and NN = G) were used as templates and subjected to PCR amplification with these 4 pairs of primers, resulting in 16 plasmids. The fidelity of all 16 monomer plasmids was confirmed by sequencing.

**Figure 3:**
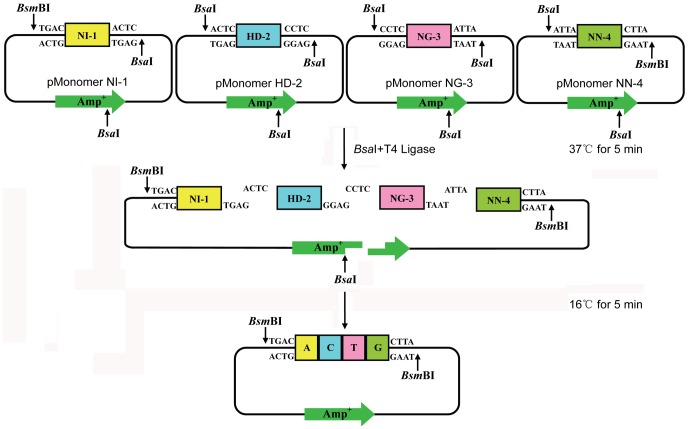
Schematic representation of the cut/ligation reaction for tetramer plasmid construction. 4 selected monomer plasmids at each position (NI-1, HD-2, NG-3 and NN-4 for example in this Figure) are put into a reaction pool containing T4 DNA ligase, *Bsa*I and ligase buffer. Once the temperature rises to 37°C, the right side of NI-1, both sides of HD-2, NG-3 and the left side of NN-4 are digested by *Bsa*I. The unique cohesive ends facilitate the positioning of each monomer in the ligation product. T4 DNA ligase joins the fragments together at 16°C. The number of constructed tetramer plasmids increases as the cycles continue.

A tetramer library including 256 possible combinations of four monomers was constructed using cut/ligation reactions based on Golden Gate cloning strategy. As *Bsa*I and *Bsm*BI have been proved to retain activity in DNA ligase buffer, digestion and ligation could be conveniently performed in one tube just by changing the temperature using a thermo cycler. We designed the tetramer with the unique structure that *Bsm*BI sites are located at 5′ end of the monomer 1 and at 3′ end of the monomer 4 and rest ends of monomers are ligated after digestion with *Bsa*I. To assemble the tetramer from the 16 monomer plasmids, we selected 4 monomer plasmids from monomer library to carry out the cut/ligate reaction in one step with *Bsa*I and T4 DNA ligase (Figure 3). The existence of a *Bsa*I site located in AMP resistance gene on pGEM-T vector improved the efficiency of tetramer construction, as this *Bsa*I site generated 3 fragments from the monomer 2 and 3 plasmids after *Bsa*I digestion, likely eliminating self-ligation of these monomer plasmids. After sequencing, we found that some colonies either had mutations introduced by PCR or contained 3 monomers. The efficiency of tetramer assembly was about 85%. The 256 tetramer library was constructed in 4 weeks.

We also constructed a trimer library which covered all 64 combinations of 3 monomers targeting 3 nucleotides by using similar approach as described above. A dimer library containing all 16 combinations of 2 monomers targeting 2 nucleotides was also constructed with the same approach.

Using these libraries (monomer, dimer, trimer and tetramer) as building blocks, we can assemble a TALE protein containing any number of monomer repeats.

### Assembling Tetramers for Construction of TALEN or TALE-TF Expression Vectors

**Figure 4:**
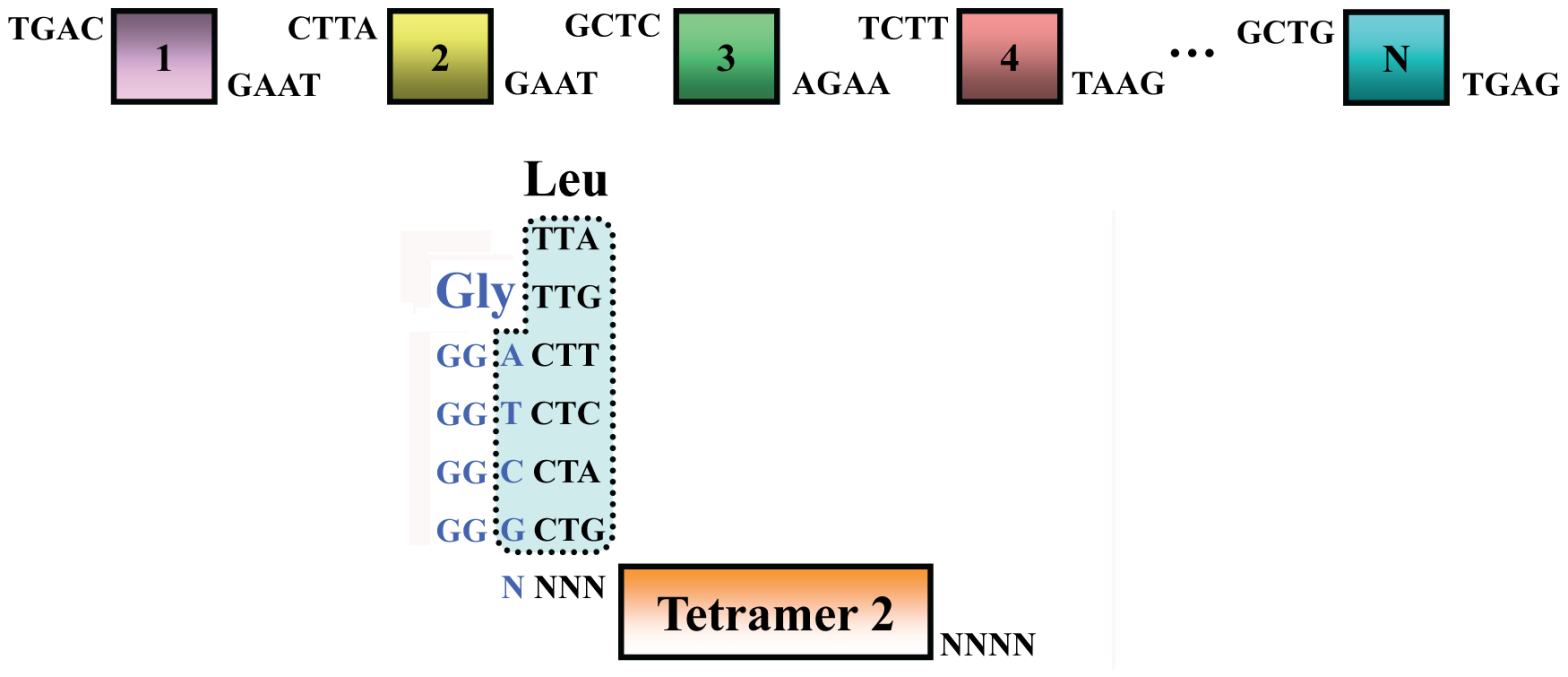
Optional assembly of TALE repeats depending on PCR mutation. Amplified with different primer pairs, repeat monomers, dimers, trimers or tetramers (1, 2, 3, 4 and N in this Figure) can be flexibly assembled in an optional order during a cut/ligation reaction. Some selected cohesive ends are shown and other possible cohesive ends can be designed by matching the last nucleotide coding Gly and three nucleotides coding Leu.

**Figure 5:**
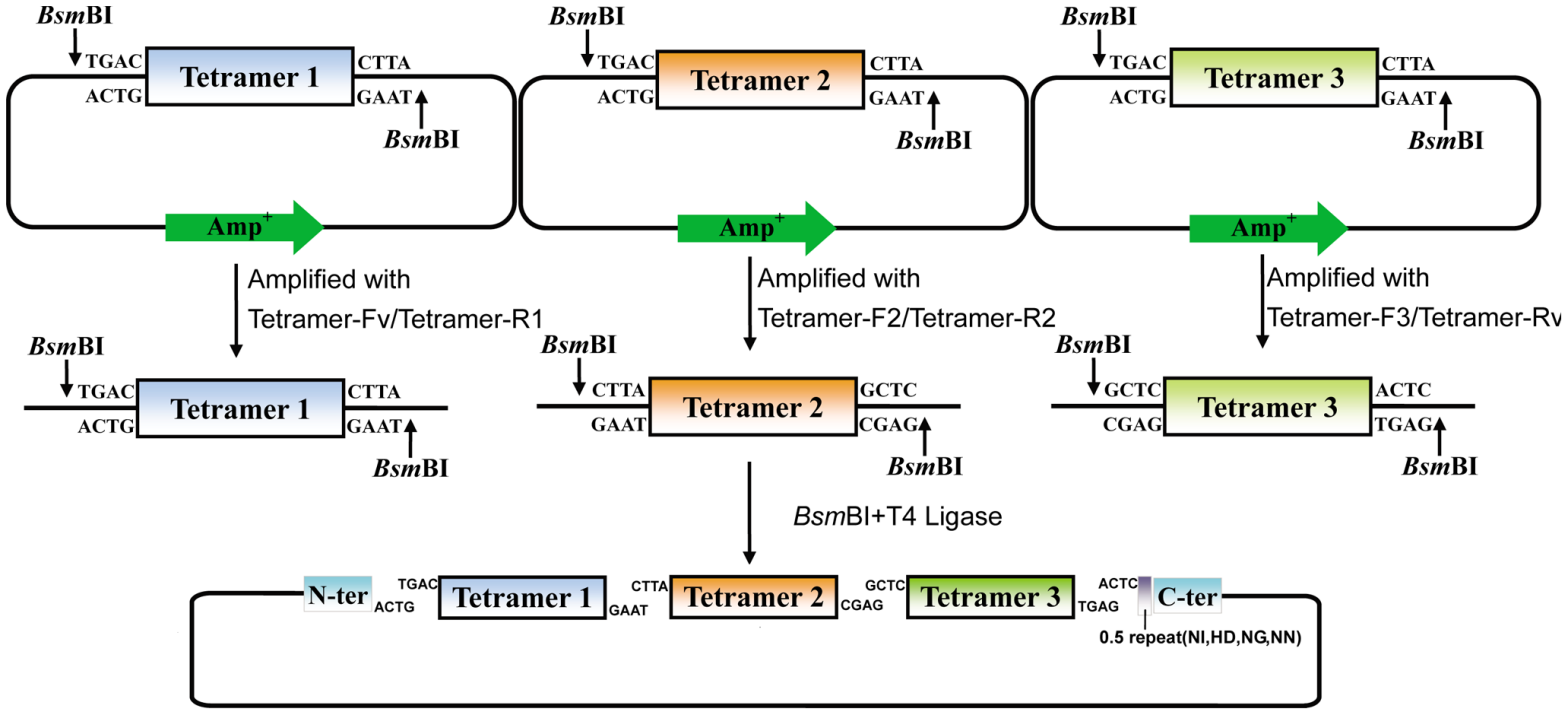
Construction of customized TALENs or TALE-TFs depending on the tetramer library. Three selected tetramer plasmids (Tetramer 1, Tetramer 2 and Tetramer 3) are amplified with separate primer pairs (Tetramer-F1/Tetramer-R1, Tetramer-F2/Tetramer-R2 and Tetramer-F3/Tetramer-R3) to be given unique cohesive ends specifying their positions in the assembly. The tetramers were then assembled and cloned into an expression backbone during a cut/ligation reaction.

All repeat tetramers in library plasmids were flanked by *Bsm*BI sites which generated TAGC at 5′ end and CTTA at 3′ end after digestion. For the assembling purpose, the tetramers were amplified by pairs of primers based on their location in TALE protein. The primers were designed to contain mismatch bases with tetramer template of library and yield a 4 base pairs unique cohesive end (the last nucleotide coding Gly and three nucleotides coding Leu) after *Bsm*BI digestion (Figure 4, Table 3). To assemble a TALE protein of any tetramer number, the first and last tetramers had to be amplified with Tetramer-Fv for the first and Tetramer-Rv for the last, since they must be compatible with vector ends. Based on this method, we assembled 3 tetramers equivalent to 12 monomer repeats (Figure 5). The 3 tetramers were selected based on target sequences. The first tetramer was amplified with Tetramer-Fv/Tetramer-R1, and the second and third tetramers were amplified with Tetramer-F2/Tetramer-R2 and Tetramer-F3/Tetramer-Rv, respectively. The PCR products were gel-purified and cut/ligation reaction was set up with the backbone vector in one tube containing *Bsm*BI/T4 ligase. Once the correct tetramers were cut out and ligated into the expression vector, the vector no longer contained any *Bsm*BI site, whereas expression vectors without inserts were linearized by *Bsm*BI during incubation for 1 h at 55°C resulting in 2 pieces to avoid false positive clones.

**Figure 6:**
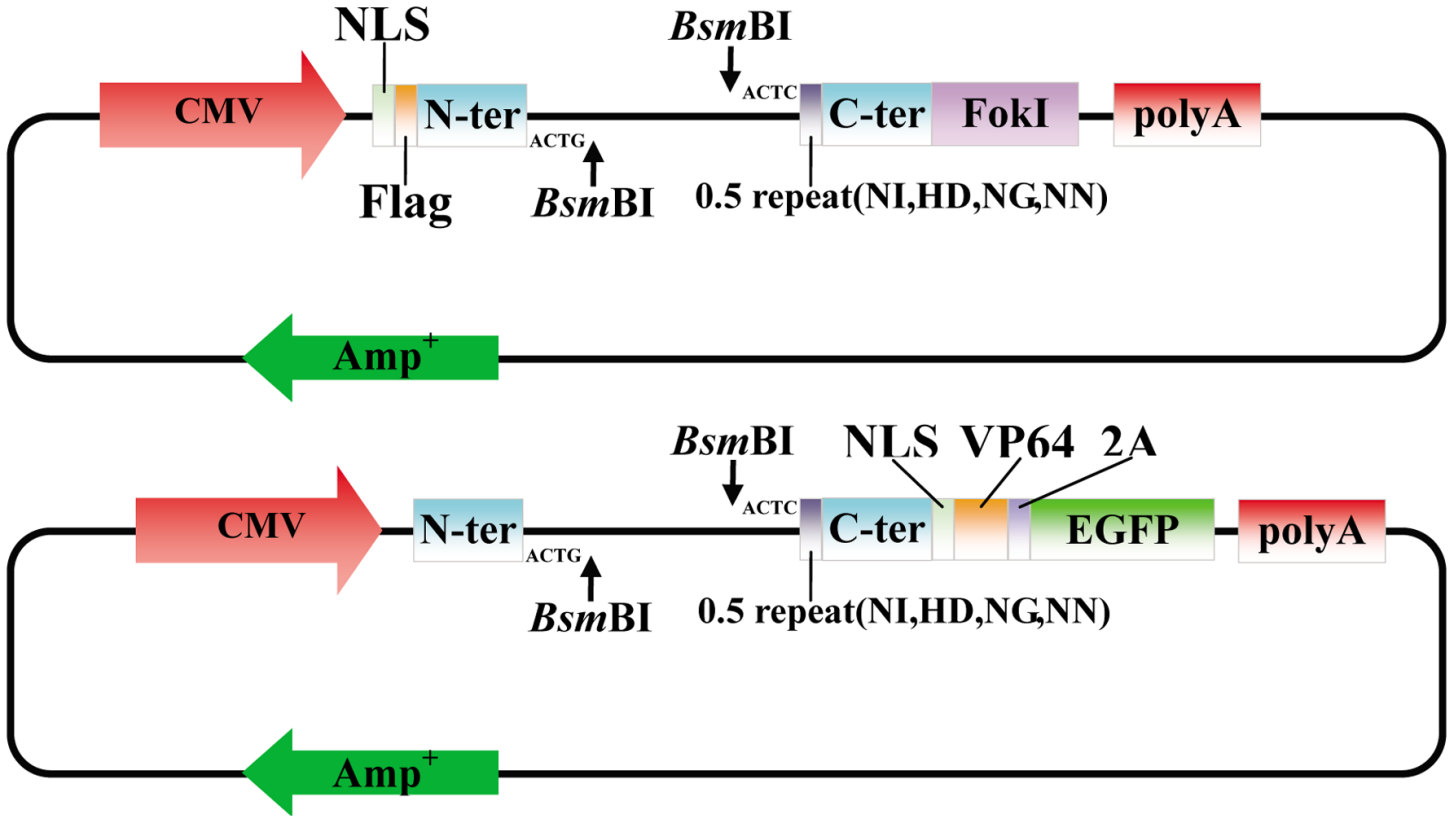
TALEN and TALE-TF expression backbones structure. The TALEN backbone contains a TALE DNA binding domain fused to the wild type FokI nuclease. The TALEN is expressed under the control of the CMV promoter. The TALE-TF backbone contains a TALE DNA binding domain fused to the VP64 transcription activator. Both the TALE-TF and EGFP are expressed under the control of the CMV promoter and they are divided by 2A. A TALEN or a TALE-TF backbone contain a 0.5 repeat that binds the final nucleotide of the targeted DNA sequence (according to the TALE binding site); thus both the TALEN backbones and TALE-TF backbones come as 4 different plasmids with 4 different 0.5 repeats.

Each of the TALEN backbones included a CMV promoter, a FLAG epitope tag, a nuclear localization signal (NLS), truncated N-terminal, C-terminal of the *Xanthomonas campestris pv. armoraciae* TALE hax3, two close *Bsm*BI sites cutting in opposite direction, a 0.5 repeat encoding one of the RVDs (NI, HD, NG or NN) and the wild-type FokI nuclease domain (Figure 6). TALE-TF backbones were pLenti-EF1a-Backbones the same as the previous study [8].

### Testing of TALENs or TALE-TFs Assembly

To test the feasibility of our method, we constructed 4 TALEN pairs targeted to mouse Gt(ROSA)26Sor gene and mouse Mstn gene sequences, and 4 TALE-TF constructs targeted to mouse Oct4, c-Myc, Klf4 and Sox2 gene promoter sequences. Binding sites were selected by using the online tool: TAL Effector-Nucleotide Targeter 2.0 (TALE-NT 2.0; https://boglab.plp.iastate.edu/) [26]. All our constructs contained 12.5 repeats. Binding sites and the lengths of spacer sequences between TALEN pair sites are shown in Table 4.

**Table 4:** TALEN constructs.

Gene	Binding site	Repeat assay	Positive rate (%)
Mouse Gt(ROSA)26Sor	TCGTGATCTGCAACtccagtctttctagaagatgggcggGAGTCTTCTGGGCA	L1: HD NN NG NN NI NG HD NG NN HD NI NI HD	60
		R1: NN HD HD HD NI NN NI NI NN NI HD NG HD	70
	TGCAACTCCAGTCTttctagaagatgggcgggagtctTCTGGGCAGGCTTA	L2: NN HD NI NI HD NG HD HD NI NN NG HD NG	60
		R2: NI NI NN HD HD NG NN HD HD HD NI NN NI	90
Mouse Mstn	TGCTGCTGGCCCAgtggatctaaatgagGGCAGTGAGAGAGA	L1: NG NN HD NG NN HD NG NN NN HD HD HD NI	55
		R1: HD NG HD NG HD NG HD NI HD NG NN HD HD	80
	TTGTGCAAATCCTGagactcatcaaacccatgaaaGACGGTACAAGGTA	L2: NG NN NG NN HD NI NI NI NG HD HD NG NN	60
		R2: NI HD HD NG NG NN NG NI HD HD NN NG HD	55

Each of the constructed TALENs or TALE-TFs was performed in 3 days prior to being sent for sequencing. For each of the 12 constructs, we picked 20 colonies for verification via colony PCR method. The positive rates of different constructs ranged from 50% to 90% (Tables 4 and 5). 153 out of 240 colonies were positive in total, with an average positive rate about 64%. 5 positive clones of each construct were sent for sequencing. All the constructs were successfully generated according to the sequencing results, although there were point mutations in few of them (Table S1). Some false clones were also sequenced (Text S2). Results indicated that addition or lack of one tetramer was the main cause, which was caused by incorrect ligation between similar adaptors (GCTC and ACTC in primer Tetramer-R2 and Tetramer-Rv which may cause tetramer 2 incorrectly ligated to the backbone). Therefore, we redesigned Tetramer-R2 and Tetramer-F3 to more divergent ones (RDT-R2 and RDT-F3 in Table 3) for efficient construction of a TALEN pair targeting sequence 5′-tccggagccccccctacctccgggggctcgcccgcgtca-3′. The positive was 80% (data not shown).

**Table 5:** TALE-TF constructs.

Gene	Binding site	Repeat assay	Positive rate (%)
Mouse Oct4	TCTCAGGGTGAATT	NG HD NG HD NI NN NN NN NG NN NI NI NG NG	65
Mouse Klf4	TGCACACGCTGCCT	NG NN HD NI HD NI HD NN HD NG NN HD HD NG	50
Mouse c-Myc	TAGAACCAATGCAC	NG NI NN NI NI HD HD NI NI NG NN HD NI HD	60
Mouse Sox2	TACCCAAGTGCATT	NG NI HD HD HD NI NI NN NG NN HD NI NG NG	60

## Discussion

Although engineering TALE protein to target specific DNA sequence is much easier than using zinc finger protein, assembling TALE repeats is technically challenging. We tried to find a rapid, cheaper and convenient method with high success rate for TALE assembly and construction, which can be quickly used by any laboratory with basic molecular biology equipment.

Golden Gate cloning strategy is a convenient and fast method for multiple fragment assembly compared with traditional cloning approaches [27]. Based on our repeat tetramer library and cut/ligation reactions, TALE constructs can be generated as quickly as other reported methods depending on the Golden Gate cloning strategy [8], [19]. Since we start from the tetramers, only one cut/ligation reaction is needed to generate TALE constructs with sufficient length of repeat arrays (12.5 repeats). We took advantage of PCR amplification to mutate the *Bsm*BI cutting sequence flanking tetramers so that only one tetramer library was needed and not three. As nothing more than fundamental molecular biology experiments, such as, PCR, gel isolation, cut-ligation and transformation, need to be performed with the most regular enzymes and facilities, methodology described here is indeed convenient and available in every laboratory.

Incorrect ligation between incompatible adaptors may be the main factor lowering the rate of positive clones. Replacement of codons on adaptors with more divergent ones may promote accurate ligations. As linearized vectors are transformed into *E. coli* competent cells accompanied with correctly constructed vectors, homology recombination between repeats may lead to incomplete repeat arrays which may increase the number of false clones. The use of PlasmidSafe exonuclease may solve this problem as previously described [19]. As PCR is used in this method, amplification errors are unavoidable. HIFI DNA polymerase can be tried in tetramer amplification to decrease mutations in further studies. The length of tandem repeats can be optionally designed according to our tetramer, trimer, dimer and monomer plasmids. We also designed some more primer pairs for assembly of longer repeat arrays in Table 3 (from Tetramer-R3 to Tetramer-F7). However, due to the limitation of the sequencing length, longer repeat arrays are not available for sequencing at present, since sequencing results longer than 700–800 bp are not credible and internal sequencing primers are not specific among repeats. Despite these limitations, the success rates prove the feasibility of this method.

## Supporting Information

Table S1 Sequencing results of positive TALE construct colonies. 5 positive colonies were sent sequencing after PCR verification and restriction digestion detection. Sequencing results information including number of correct colonies, number of point mutant colonies and number of point mutant colonies were shown in the table.
(DOC)

Text S1 Sequencing result of a correct Gt(ROSA)26Sor-L1 colony. 5 positive colonies of Gt(ROSA)26Sor-L1 were sent sequencing. Sequencing result of one out of four correct colonies were given in the text.
(DOC)

Text S2 Sequencing result of a tetramer lost Gt(ROSA)26Sor-L1 colony. A false colony of Gt(ROSA)26Sor-L1 was sent sequencing. Sequencing result presented in the text indicated that the last repeat tetramer between adaptor GCTC and ACTC was lost. The adaptor became ACTC and was marked in red.
(DOC)

## References

[1] Pennisi E (2012) The tale of the TALEs. Science 338: 1408–1411. 23239709 10.1126/science.338.6113.1408

[2] Kay S, Hahn S, Marois E, Hause G, Bonas U (2007) A bacterial effector acts as a plant transcription factor and induces a cell size regulator. Science 318: 648–651. 17962565 10.1126/science.1144956

[3] Romer P, Hahn S, Jordan T, Strauss T, Bonas U, et al (2007) Plant pathogen recognition mediated by promoter activation of the pepper Bs3 resistance gene. Science 318: 645–648. 17962564 10.1126/science.1144958

[4] Boch J, Scholze H, Schornack S, Landgraf A, Hahn S, et al (2009) Breaking the code of DNA binding specificity of TAL-type III effectors. Science 326: 1509–1512. 19933107 10.1126/science.1178811

[5] Moscou MJ, Bogdanove AJ (2009) A simple cipher governs DNA recognition by TAL effectors. Science 326: 1501. 19933106 10.1126/science.1178817

[6] Cermak T, Doyle EL, Christian M, Wang L, Zhang Y, et al (2011) Efficient design and assembly of custom TALEN and other TAL effector-based constructs for DNA targeting. Nucleic Acids Res 39: e82. 21493687 10.1093/nar/gkr218 PMC3130291

[7] Ma S, Zhang S, Wang F, Liu Y, Xu H, et al (2012) Highly efficient and specific genome editing in silkworm using custom TALENs. PLoS One 7: e45035. 23028749 10.1371/journal.pone.0045035 PMC3445556

[8] Zhang F, Cong L, Lodato S, Kosuri S, Church GM, et al (2011) Efficient construction of sequence-specific TAL effectors for modulating mammalian transcription. Nat Biotechnol 29: 149–153. 21248753 10.1038/nbt.1775 PMC3084533

[9] Bultmann S, Morbitzer R, Schmidt CS, Thanisch K, Spada F, et al (2012) Targeted transcriptional activation of silent oct4 pluripotency gene by combining designer TALEs and inhibition of epigenetic modifiers. Nucleic Acids Res 40: 5368–5377. 22387464 10.1093/nar/gks199 PMC3384321

[10] Li T, Huang S, Zhao X, Wright DA, Carpenter S, et al (2011) Modularly assembled designer TAL effector nucleases for targeted gene knockout and gene replacement in eukaryotes. Nucleic Acids Res 39: 6315–6325. 21459844 10.1093/nar/gkr188 PMC3152341

[11] Sander JD, Cade L, Khayter C, Reyon D, Peterson RT, et al (2011) Targeted gene disruption in somatic zebrafish cells using engineered TALENs. Nat Biotechnol 29: 697–698. 21822241 10.1038/nbt.1934 PMC3154023

[12] Huang P, Xiao A, Zhou M, Zhu Z, Lin S, et al (2011) Heritable gene targeting in zebrafish using customized TALENs. Nat Biotechnol 29: 699–700. 21822242 10.1038/nbt.1939

[13] Dahlem TJ, Hoshijima K, Jurynec MJ, Gunther D, Starker CG, et al (2012) Simple methods for generating and detecting locus-specific mutations induced with TALENs in the zebrafish genome. PLoS Genet 8: e1002861. 22916025 10.1371/journal.pgen.1002861 PMC3420959

[14] Young JJ, Cherone JM, Doyon Y, Ankoudinova I, Faraji FM, et al (2011) Efficient targeted gene disruption in the soma and germ line of the frog Xenopus tropicalis using engineered zinc-finger nucleases. Proc Natl Acad Sci U S A 108: 7052–7057. 21471457 10.1073/pnas.1102030108 PMC3084115

[15] Tesson L, Usal C, Menoret S, Leung E, Niles BJ, et al (2011) Knockout rats generated by embryo microinjection of TALENs. Nat Biotechnol 29: 695–696. 21822240 10.1038/nbt.1940

[16] Miller JC, Tan S, Qiao G, Barlow KA, Wang J, et al (2011) A TALE nuclease architecture for efficient genome editing. Nat Biotechnol 29: 143–148. 21179091 10.1038/nbt.1755

[17] Mussolino C, Morbitzer R, Lutge F, Dannemann N, Lahaye T, et al (2011) A novel TALE nuclease scaffold enables high genome editing activity in combination with low toxicity. Nucleic Acids Res 39: 9283–9293. 21813459 10.1093/nar/gkr597 PMC3241638

[18] Hockemeyer D, Wang H, Kiani S, Lai CS, Gao Q, et al (2011) Genetic engineering of human pluripotent cells using TALE nucleases. Nat Biotechnol 29: 731–734. 21738127 10.1038/nbt.1927 PMC3152587

[19] Sanjana NE, Cong L, Zhou Y, Cunniff MM, Feng G, et al (2012) A transcription activator-like effector toolbox for genome engineering. Nat Protoc 7: 171–192. 22222791 10.1038/nprot.2011.431 PMC3684555

[20] Geissler R, Scholze H, Hahn S, Streubel J, Bonas U, et al (2011) Transcriptional activators of human genes with programmable DNA-specificity. PLoS One 6: e19509. 21625585 10.1371/journal.pone.0019509 PMC3098229

[21] Morbitzer R, Elsaesser J, Hausner J, Lahaye T (2011) Assembly of custom TALE-type DNA binding domains by modular cloning. Nucleic Acids Res 39: 5790–5799. 21421566 10.1093/nar/gkr151 PMC3141260

[22] Weber E, Gruetzner R, Werner S, Engler C, Marillonnet S (2011) Assembly of designer TAL effectors by Golden Gate cloning. PLoS One 6: e19722. 21625552 10.1371/journal.pone.0019722 PMC3098256

[23] Li L, Piatek MJ, Atef A, Piatek A, Wibowo A, et al (2012) Rapid and highly efficient construction of TALE-based transcriptional regulators and nucleases for genome modification. Plant Mol Biol 78: 407–416. 22271303 10.1007/s11103-012-9875-4 PMC3580834

[24] Reyon D, Tsai SQ, Khayter C, Foden JA, Sander JD, et al (2012) FLASH assembly of TALENs for high-throughput genome editing. Nat Biotechnol 30: 460–465. 22484455 10.1038/nbt.2170 PMC3558947

[25] Briggs AW, Rios X, Chari R, Yang L, Zhang F, et al (2012) Iterative capped assembly: rapid and scalable synthesis of repeat-module DNA such as TAL effectors from individual monomers. Nucleic Acids Res 40: e117. 22740649 10.1093/nar/gks624 PMC3424587

[26] Doyle EL, Booher NJ, Standage DS, Voytas DF, Brendel VP, et al (2012) TAL Effector-Nucleotide Targeter (TALE-NT) 2.0: tools for TAL effector design and target prediction. Nucleic Acids Res 40: W117–122. 22693217 10.1093/nar/gks608 PMC3394250

[27] Engler C, Kandzia R, Marillonnet S (2008) A one pot, one step, precision cloning method with high throughput capability. PLoS One 3: e3647. 18985154 10.1371/journal.pone.0003647 PMC2574415

