# Supplementary material for: A Simple and Efficient Method for Assembling TALE Protein Based on Plasmid Library
Source: PLoS One. 2013 Jun 20;8(6):e66459. doi: 10.1371/journal.pone.0066459 (PMC3688977; doi:10.1371/journal.pone.0066459)
Supplement: Table S1 — Sequencing results of positive TALE construct colonies. 5 positive colonies were sent sequencing after PCR verification and restriction digestion detection. Sequencing results information including number of correct colonies, number of point mutant colonies and number of point mutant colonies were shown in the table. (DOC) [file pone.0066459.s001.doc]

Table S1. Sequencing results of positive TALE construct colonies.

| TALE construct | Number of colonies sequenced | Number of correct colonies | Number of point mutant colonies | Correction rate (%) |
| --- | --- | --- | --- | --- |
| Gt(ROSA)26Sor-L1 | 5 | 4 | 1 | 80 |
| Gt(ROSA)26Sor-R1 | 5 | 5 | 0 | 100 |
| Gt(ROSA)26Sor-L2 | 5 | 5 | 0 | 100 |
| Gt(ROSA)26Sor-R2 | 5 | 5 | 0 | 100 |
| Mstn-L1 | 5 | 4 | 1 | 80 |
| Mstn-R1 | 5 | 5 | 0 | 100 |
| Mstn-L2 | 5 | 5 | 0 | 100 |
| Mstn-R2 | 5 | 4 | 1 | 80 |
| TALE-Oct4 | 5 | 5 | 0 | 100 |
| TALE-Klf4 | 5 | 4 | 1 | 80 |
| TALE-cMyc | 5 | 4 | 1 | 80 |
| TALE-Sox2 | 5 | 5 | 0 | 100 |
