# Supplementary material for: A Simple and Efficient Method for Assembling TALE Protein Based on Plasmid Library
Source: PLoS One. 2013 Jun 20;8(6):e66459. doi: 10.1371/journal.pone.0066459 (PMC3688977; doi:10.1371/journal.pone.0066459)
Supplement: Text S1 — Sequencing result of a correct Gt(ROSA)26Sor-L1 colony. 5 positive colonies of Gt(ROSA)26Sor-L1 were sent sequencing. Sequencing result of one out of four correct colonies were given in the text. (DOC) [file pone.0066459.s002.doc]

Text S1. Sequencing result of a correct Gt(ROSA)26Sor-L1 colony

AAGCGACGACGCTGAGGCCCTGTTGACGGTCGCGGGAGAGCTGAGAGGGCCTCCCCTTCAGCTGGACACGGGCCAGTTGCTGAAGATCGCGAAGCGGGGAGGAGTCACGGCGGTCGAGGCGGTGCACGCGTGGCGCAATGCGCTCACGGGAGCACCCCTCAACCTGACCCCAGAGCAGGTCGTGGCGATCGCAAGCCACGACGGAGGAAAGCAAGCCTTGGAAACAGTACAGAGGCTGTTGCCTGTGCTGTGCCAAGCGCACGGACTCACCCCAGAGCAGGTCGTGGCAATCGCGAGCAATAACGGCGGAAAACAGGCTTTGGAAACGGTGCAGAGGCTCCTTCCAGTGCTGTGCCAAGCGCACGGCCTCACCCCAGAGCAGGTCGTGGCCATTGCCTCGAATGGAGGGGGCAAACAGGCGTTGGAAACCGTACAACGATTGCTGCCGGTGCTGTGCCAAGCGCACGGATTAACCCCAGAGCAGGTCGTGGCAATCGCGAGCAATAACGGCGGAAAACAGGCTTTGGAAACGGTGCAGAGGCTCCTTCCAGTGCTGTGCCAAGCGCACGGCTTAACCCCAGAGCAGGTCGTGGCAATCGCCTCCAACATTGGCGGGAAACAGGCACTCGAGACTGTCCAGCGCCTGCTTCCCGTGCTGTGCCAAGCGCACGGACTCACCCCAGAGCAGGTCGTGGCCATTGCCTCGAATGGAGGGGGCAAACAGGCGTTGGAAACCGTACAACGATTGCTGCCGGTGCTGTGCCAAGCGCACGGCCTCACCCCAGAGCAGGTCGTGGCGATCGCAAGCCACGACGGAGGAAAGCAAGCCTTGGAAACAGTACAGAGGCTGTTGCCTGTGCTGTGCCAAGCGCACGGATTAACCCCAGAGCAGGTCGTGGCCATTGCCTCGAATGGAGGGGGCAAACAGGCGTTGGAAACCGTACAACGATTGCTGCCGGTGCTGTGCCAAGCGCACGGGCTTACCCCAGAGCAGGTCGTGGCAATCGCGAGCAATAACGGCGGAAAACAGGCTTTGGAAACGGTGCAGAGGCTCCTTCCAGTGCTGTGCCAAGCGCACGGACTCACCCCAGAGCAGGTCGTGGCGATCGCAAGCCACGACGGAGGAAAGCAAGCCTTGGAAACAGTACAGAGGCTGTTGCCTGTGCTGTGCCAAGCGCACGGCCTCACCCCAGAGCAGGTCGTGGCAATCGCCTCCAACATTGGCGGGAAACAGGCACTCGAGACTGTCCAGCGCCTGCTTCCCGTGCTGTGCCAAGCGCACGGATTAACCCCAGAGCAGGTCGTGGCAATCGCCTCCAACATTGGCGGGAAACAGGCACTCGAGACTGTCCAGCGCCTGCTTCCCGTGCTGTGCCAAGCGCACGGACTCACGCCTGAGCAGGTAGTGGCTATTGCATCCCATGACGGGGGCAGACCCGCACTGGATCAATCTGCAGAATCTTCTT
