# Supplementary material for: A Simple and Efficient Method for Assembling TALE Protein Based on Plasmid Library
Source: PLoS One. 2013 Jun 20;8(6):e66459. doi: 10.1371/journal.pone.0066459 (PMC3688977; doi:10.1371/journal.pone.0066459)
Supplement: Text S2 — Sequencing result of a tetramer lost Gt(ROSA)26Sor-L1 colony. A false colony of Gt(ROSA)26Sor-L1 was sent sequencing. Sequencing result presented in the text indicated that the last repeat tetramer between adaptor GCTC and ACTC was lost. The adaptor became ACTC and was marked in red. (DOC) [file pone.0066459.s003.doc]

Text S2. Sequencing result of a tetramer lost Gt(ROSA)26Sor-L1 colony

CGACGACGCTGAGGCCTGTTGACGGTCGCGGGAGAGCTGAGAGGGCCTCCCCTTCAGCTGGACACGGGCCAGTTGCTGAAGATCGCGAAGCGGGGAGGAGTCACGGCGGTCGAGGCGGTGCACGCGTGGCGCAATGCGCTCACGGGAGCACCCCTCAACCTGACCCCAGAGCAGGTCGTGGCGATCGCAAGCCACGACGGAGGAAAGCAAGCCTTGGAAACAGTACAGAGGCTGTTGCCTGTGCTGTGCCAAGCGCACGGACTCACCCCAGAGCAGGTCGTGGCAATCGCGAGCAATAACGGCGGAAAACAGGCTTTGGAAACGGTGCAGAGGCTCCTTCCAGTGCTGTGCCAAGCGCACGGCCTCACCCCAGAGCAGGTCGTGGCCATTGCCTCGAATGGAGGGGGCAAACAGGCGTTGGAAACCGTACAACGATTGCTGCCGGTGCTGTGCCAAGCGCACGGATTAACCCCAGAGCAGGTCGTGGCAATCGCGAGCAATAACGGCGGAAAACAGGCTTTGGAAACGGTGCAGAGGCTCCTTCCAGTGCTGTGCCAAGCGCACGGCTTAACCCCAGAGCAGGTCGTGGCAATCGCCTCCAACATTGGCGGGAAACAGGCACTCGAGACTGTCCAGCGCCTGCTTCCCGTGCTGTGCCAAGCGCACGGACTCACCCCAGAGCAGGTCGTGGCCATTGCCTCGAATGGAGGGGGCAAACAGGCGTTGGAAACCGTACAACGATTGCTGCCGGTGCTGTGCCAAGCGCACGGCCTCACCCCAGAGCAGGTCGTGGCGATCGCAAGCCACGACGGAGGAAAGCAAGCCTTGGAAACAGTACAGAGGCTGTTGCCTGTGCTGTGCCAAGCGCACGGATTAACCCCAGAGCAGGTCGTGGCCATTGCCTCGAATGGAGGGGGCAAACAGGCGTTGGAAACCGTACAACGATTGCTGCCGGTGCTGTGCCAAGCGCACGGACTCACGCCTGAGCAGGTAGTGGCTATTGCATCCCATGACGGGGGCAGACCCGCACTGGAGTCATCTGCAGACC
